# Retraction: Enhanced Immune Response and Protective Effects of Nano-chitosan-based DNA Vaccine Encoding T Cell Epitopes of Esat-6 and FL against *Mycobacterium Tuberculosis* Infection

**DOI:** 10.1371/journal.pone.0303549

**Published:** 2024-05-08

**Authors:** The PLOS ONE Editors

After this article [1] was published, concerns were raised about Figs 1B, 5C, S3, and S4.

Specifically:

There appear to be similarities between panels within Figs 1B, S3, and S4 of this article [1]. The corresponding authors have indicated that there were errors in the preparation of these figures:

- In Fig 1B, lanes 1–3 of the anti-His panel look similar to lanes 2–4 of the anti-FL panel.
- In Fig S3, the left-hand region of the nano-chitosan-GFP(48h) panel appears similar to the right-hand region of the pGCsi-GFP(72h) panel.
- In Fig S4A of [1], the upper right region of the nano-pIRES panel appears similar to the upper left region of the nano-FL panel when rotated 90 degrees.

There appear to be similarities between panels in Figs 5C and S4 of this article [1] and panels in Fig 7 of another article by the same authors [2] before its correction in [3]. The corresponding authors have indicated that the images are correctly used in this article [1], but incorrectly used in [2], with the error subsequently corrected in [3]:

- The nano-Esat-6-FL panel in Fig 5C of [1] appears similar to the DNA/Ags pIRES-EPS-FL panel in Fig 7C of [2] before its correction in [3].
- The nano-pIRES panel in Fig S4A of [1] appears similar to the DNA/Ags PBS panel of Fig 7E of [2] before its correction in [3].
- The nano-Esat-6/3e-FL panel of Fig S4A of [1] appears similar to the DNA/Ags pIRES-EPS-FL panel of Fig 7E of [2] before its correction in [3].
- The upper right region of the nano-Esat-6/3e-FL panel of Fig S4B of [1] appears similar to the lower left region of the DNA pIRES-EPS-FL panel of Fig 7F of [2] before its correction in [3].
- The Esat-6/3e-FL panel of Fig S4B of [1] appears similar to the DNA/Ags pIRES-EPS panel of Fig 7F of [2] before its correction in [3].

The corresponding authors provided replacement images from independent experimental replicates, and for Figs 1B and S4 they also provided cropped versions of the images in the published figures. However, they stated that the original uncropped and unadjusted images underlying the above-mentioned published figures are no longer available; as such, the concerns about the published figures could not be resolved.

In light of the extent of the above issues, which raise concerns about the reliability of the published results, the *PLOS ONE* Editors retract this article.

GF and YW did not agree with the retraction and stand by the article’s findings. QJ, MX, YL, WQ, DZ, LL, and GP either did not respond directly or could not be reached.

## References

[1] Feng G, Jiang Q, Xia M, Lu Y, Qiu W, Zhao D, et al. (2013) Enhanced Immune Response and Protective Effects of Nano-chitosan-based DNA Vaccine Encoding T Cell Epitopes of Esat-6 and FL against *Mycobacterium Tuberculosis* Infection. PLoS ONE 8(4): e61135. 10.1371/journal.pone.0061135 23637790 PMC3634041

[2] Jiang Q, Zhang J, Chen X, Xia M, Lu Y, et al. (2013) A novel recombinant DNA vaccine encoding Mycobacterium tuberculosis ESAT-6 and FL protects against Mycobacterium tuberculosis challenge in mice. The Journal of Biomedical Research 27(5): 406–420. 10.7555/JBR.27.20120114 24086174 PMC3783826

[3] Jiang Q, Zhang J, Chen X, Xia M, Lu Y, et al. (2022) Author Correction: A novel recombinant DNA vaccine encoding Mycobacterium tuberculosis ESAT-6 and FL protects against Mycobacterium tuberculosis challenge in mice. The Journal of Biomedical Research 36(6):448–450 36642894 PMC9724156

